# Does implant removal of superior clavicle plate osteosynthesis affect the functional outcome: a prospective trial

**DOI:** 10.1007/s00402-020-03669-z

**Published:** 2020-11-01

**Authors:** Markus Wurm, Marc Beirer, Michael Zyskowski, Christopher Völk, Arthur Schwarz, Peter Biberthaler, Chlodwig Kirchhoff, Moritz Crönlein

**Affiliations:** Department of Trauma Surgery, Klinikum Rechts Der Isar, Technical University of Munich, Ismaninger Strasse 22, 81675 Munich, Germany

**Keywords:** Implant removal, Clavicle fractures, Hardware removal, Elective surgery, Disturbing implants, Irritation

## Abstract

**Background:**

Elective implant removal (IR) accounts for up to 30% of all orthopaedic surgeries. While there is general acceptance about the need of implant removal for obvious reasons, such as infections or implant failure, little is known about the beneficial aspects in cases of minor reasons such as patients’ wish for IR. Therefore, we initiated this study to define patients’ benefit of elective implant removal following plate osteosynthesis of displaced clavicle fractures.

**Patients and methods:**

Prospective evaluation of patients was conducted before implant removal and 6 weeks postoperative. Subjective and objective criteria included pain rating on a visual analogue scale (VAS) and active range of motion (ROM) pre- and 6 weeks postoperative. Functional scoring included Constant-Murley Score, DASH (Disabilities of Arm, Shoulder and Hand Score), MSQ (Munich Shoulder Questionnaire) and SPADI (Shoulder Pain and Disability Index).

**Results:**

37 patients were prospectively enrolled in this study and implant removal was performed after 16 ± 6.1 months. No re-fractures nor other complications were detected during routine follow up. Functional outcome increased through all scores (Constant score 73.3 ± 14.6 preoperative to 87.4 ± 12.0 postoperative (*p* = 0.000), MSQ 85.0 ± 7.3 preoperative to 91.8 ± 9.0 postoperative (*p* = 0.005), DASH Score 7.4 ± 8.2 preoperative to 5.7 ± 9.5 postoperative (*p* = 0.414), SPADI 93.4 ± 6.6 preoperative to 94.0 ± 10.1 postoperative (*p* = 0.734).

**Conclusions:**

Discomfort during daily activities or performing sports as well as limited range of motion were the main reasons for patients’ wish for implant removal. We found increased functional outcome parameters and decreased irritation after implant removal. Therefore we suggest implant removal in case of patients’ wish and completed fracture consolidation.

**Trial registration:**

Trial registration no: NCT04343118, Retrospective registered: www.clinicaltrials.gov.

## Background

Treatment of displaced clavicle fractures changed remarkably over the last decades towards operative regimens [1]. Displaced clavicle fractures can be treated using elastic stable intramedullary nails (ESIN) or plate osteosynthesis [2, 3]. Newly developed implants, combining a variable angle stability with a low profile anatomically pre-shaped plate design show promising results in modern fracture care using open reduction and internal fixation (ORIF) [4, 5]. However, there are still patients complaining about disturbing implants, limited range of motion or weather dependent pain even though no radiological deficiencies can be detected [6–8]. While numerous reasons for patients’ complaints would be conceivable, the implanted material itself is meant to cause certain problems in many cases [9].

Obvious reasons for the need of implant removal in general, such as infections, implant loosening or implant associated nerve lesions, have been described by few authors and gain worldwide acceptance [10, 11]. However, elective implant removal for minor reasons, such as limited range of motion or a disturbing implant, is not proven to be beneficial overall [12, 13]. Implant removal accounts for up to 30% of all elective orthopaedic surgeries and rate of irritation followed by patients’ wish for IR is increasing [8, 9, 14], even though there is a clear lack of evidence about the substantial need of implant removal in consolidated fractures [15].

Being aware of patients’ complaints about disturbing implants, especially in exposed body parts as the shoulder girdle, we initiated this prospective study to elucidate reasons for patients’ wish of implant removal as well as patients’ benefit after elective implant removal following plate osteosynthesis of displaced clavicle fractures.

## Material and methods

### Patients

154 patients were operatively treated for displaced clavicle fractures in our level 1 trauma center between July 1st 2012 and July 1st 2015. 21 patients received elastic stable intramedullary nail (ESIN) implantation and 133 patients plate osteosynthesis, respectively. Patients who received ESIN were excluded from this study to rule out implant related bias. All patients fulfilling inclusion criteria during this period were prospectively enrolled and asked to participate in this trial in case of wish for implant removal. 37 (27.8%) patients asked for implant removal due to pain, irritation and restriction during daily activities and sports, respectively. 7 patients were lost to follow up and 3 patients did not complete all follow up questionnaires why they were excluded from statistical workup. Overall 27 patients fulfilled inclusion criteria and completed all follow ups.

Inclusion criteria were all patients (> 18 years) who presented in our outpatient clinic with radiologically consolidated clavicle fractures following ORIF using a superior anatomically preformed locking plate, who asked for elective implant removal due to disturbing plate material, limited range of motion (ROM) or weather dependent pain, were prospectively enrolled in our study after giving written informed consent. Excluded from this study were pregnant patients, as well as under-aged or delinquent patients.

Institutional Review Board was obtained by competent Ethical Committee (IRB approval No: 99/17S, Ethical committee Technical University Munich). Trial registration was retrospectively performed (NCT04343118). Statistical analysis was performed using SPSS 25 for MAC (Chicago, IL, USA). Power analysis was performed prior to this study using G*Power for Mac. Wilcoxon signed-rank test was used to compare means of pre and postoperative values. The significance threshold was set at a *p* value of < 0.05.

### Surgery and aftercare

**Fig. 1:**
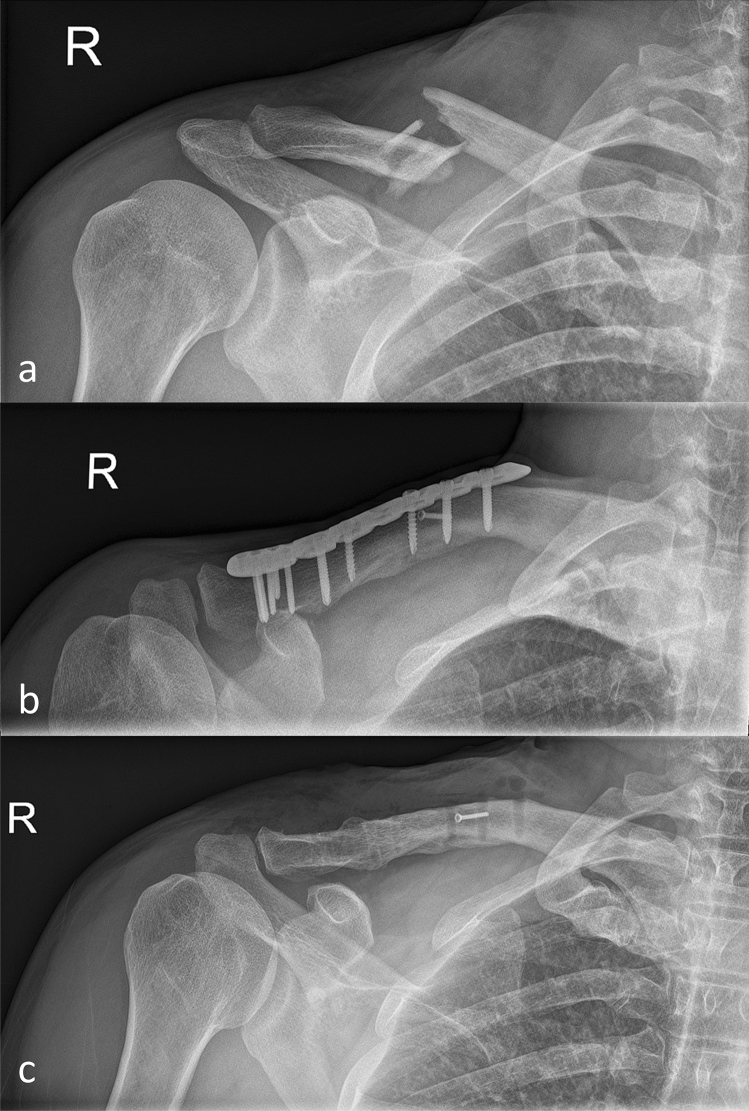
Midshaft clavicle fracture (OTA 15.C2) preoperative (**a**), after osteosynthesis using a preformed distal end clavicle plate (Arthrex ®) (**b**) and after implant removal with remaining 2.0 mm cortical screw (**c**)

All patients presented with radiologically consolidated clavicle fractures following plate osteosynthesis. General anaesthesia was used in all cases and a single dose of 1.5 mg cephalosporin was given preoperatively for prophylaxis. All patients were positioned in beach chair position on a radiolucent table. The initial superior approach to the clavicle was utilized in all cases. Early active motion without restrictions started one day postoperatively. Sporting activities (i.e. jogging etc.) were allowed six weeks postoperatively (Fig. 1).

### Evaluation

Personal interviews and shoulder scoring were carried out by an independent investigator (AS) not involved in the operative treatment or later statistical processing. All patients gave informed consent prior to being included to the study. The evaluation of the patients was conducted preoperatively and 6 weeks postoperatively during consultation in our outpatient clinic. Patients were asked for the main reason for their wish of IR. For subjective functional examination, the MSQ was handed out to all patients at both times of evaluation [16, 17]. Objective assessment consisted of a physical examination for active shoulder ROM. Moreover, sensomotoric disturbances and postoperative complications were reviewed. Functional scoring included a visual analogue scale (VAS) for pain rating, SPADI, DASH score, the Constant-Murley Shoulder Outcome Score (Constant Score) and the MSQ.

## Results

Overall 37 consecutive patients (32 male, 5 female) with a mean age of 43.3 ± 12.7 years were enrolled. Except three, all other patients fulfilled inclusion criteria and all follow ups were completed yet seven patients were lost to follow up and were therefore excluded. Overall 27 patients were included for final statistical processing Table 1.

**Table 1:** Fracture location utilizing AO/OTA classification for midshaft and Jäger&Breitner classification for distal end clavicle fractures

AO/OTA classification	15-B1	15-B2	15-B3
	6	6	10
Jäger&Breitner classification	Type IIa	Type IIb	Type III
	4	0	1

Implant removal was performed after 16 ± 6.1 months. Mean duration of initial ORIF was 92 ± 28 min. Duration of implant removal was significantly faster (49 ± 17 min; *p* = 0.000) compared to initial ORIF. We did not encounter re-fractures in this cohort throughout the follow up examinations. No wound healing disorders or infections were detected either. The mean Constant-Murley score increased from 73.3 ± 14.6 preoperative to 87.4 ± 12.0 postoperative [*p* = 0.000, statistical significant (s.)]. MSQ improved from 85.0 ± 7.3 preoperative to 91.8 ± 9.0 postoperative (*p* = 0.005, s.). Mean DASH Score improved from 7.4 ± 8.2 preoperative to 5.7 ± 9.5 postoperative [*p* = 0.414, not statistical significant (n.s.)] as well as SPADI did from 93.4 ± 6.6 preoperative to 94.0 ± 10.1 postoperative (*p* = 0.734, n.s.), respectively Table 2.

**Table 2:** Main reason for patients’ wish for implant removal after ORIF of dislocated clavicle fracture

Main reason for implant removal	*n* = (*X* %)
Pain overall	11 (40.7%)
Restriction/Irritation during daily activities	8 (29.6%)
Restriction/Irritation during sportive activities	6 (22.2%)
Others	2 (7.4%)

Subitem analysis was performed to identify certain risk factors which led to patients’ wish for IR. Eleven patients (40.7%) reported pain to be the main reason for their wish for IR. Further reasons were restriction in daily activities (*n* = 8; 29.6%), restriction during sportive activities (*n* = 6; 22.2%) and other reasons in 2 patients (7.4%). Patients reported on irritation or pain during daily activities and recreational sports. Pre and postoperative mean pain levels during the day revealed significant improvement with a mean pain level (VAS) of 1.4 (*p* = 0.028, s.). After IR mean pain levels during sleeping decreased significantly (*p* = 0.018, s.). Patients were asked how often they ponder about their implant and/or limited ROM. We could detect a significant improvement in this item after implant removal utilizing the MSQ from preoperative 8.2 postoperative 8.9 (*p* = 0.019, s.). Best improvement with respect to degrees of ROM was detected for internal rotation (*p* = 0.101, n.s.) yet without statistical significance. Abduction, (*p* = 0.561, n.s.), external rotation (*p* = 0.000, s.) and flexion (*p* = 0.468, n.s.) only showed less improvement. External rotation showed statistical significant values yet the actual degrees of advancement in ROM was less compared to internal rotation.

## Discussion

Surgical treatment of clavicle fractures gained importance over the last two decades. The validated Swedish Hospital Discharge Register revealed an increase of 705% operatively treated clavicle fractures between 2001 and 2012 [1]. According to these numbers, a concomitant rise on implant removal can be expected for the future. The German Speaking society for Orthopedics and Traumatology (DGOU) consented no obligate need for implant removal in 2018 [15]. Therefore, our goal was to gather information with regards of substantial need of implant removal after ORIF of dislocated clavicle fractures. While literature grew over the past years with respect to this topic, there is still a lack of information about clear indication as well as clinical and functional outcome after IR.

In 2007 Minkowitz et al. reported on implant removal after orthopedic fracture treatment as a safe procedure with minimal risk and good functional outcome in various surgical sites [18]. Williams et al. reported on excellent outcomes after implant removal in patients with ankle fractures. They further state implant removal as a common procedure in foot and ankle surgery due to pain and patients’ wish which goes along with other authors [18–20]. While implant removal is mainly performed due to pain in foot and ankle surgery, impaired function and irritation were the main reasons for patients’ wish of implant removal in our cohort. Snoddy et al. also reported on pain (30%) as the main factor for implant removal after distal radius fracture [21].

All patients included in our study requested for implant removal due to complaints or pain during daily activities or sports respectively. While pain was the most common finding in the presented cohort Hulsmans et al. found irritation to be reason number one for IR [8]. No mal or non-union occurred in this cohort during given follow up period. We suspected superior plate positioning could be one conceivable reason for patients` complaints, yet Hulsmans et al. revealed equal irritation rates after superior and anterior plate positioning [22]. Wang et al. reported on a 96% rate of implant removals after osteosynthesis of the clavicle. Their recommendation goes along with our approach to only perform implant removal after patients’ explicit request [23]. Another important issue is informed patients’ consent since remnants of implanted material can be left in the clavicle due to technical complications i.e. screw breakage etc. [24]. However, from our point of view additional harm should be avoided to prevent decreased bony stability.

Future research and interest could furthermore be directed towards another issue which also led to this study as we considered the chance of “psychologic relieve” after implant removal. Therefore patients were asked for pondering about sustained injury and implanted material as factor which causes limitation and pain. Since we found statistical significant improvement (*p* = 0.019, s.) for this subject we think the circumstance of implanted material itself can cause limitations, which would suggest implant removal to gain or even restore normal shoulder function.

Our results revealed improvement in all levels of ROM yet without statistical significant values. Furthermore decreased pain levels comparable to other research groups where found [8, 23]. Due to an exceptional reported rise of operatively treated clavicle fractures, we assume accompanied rise of implant related irritations going along with the patients’ wish for implant removal. Our reported outcomes reveal improvement of shoulder function in daily activities (*p* = 0.064, n.s.) and sports (*p* = 0.219, n.s.) after IR. Over 40% of presented patients reported pain being the main reason why they were distracted and restricted during daily life. Therefore implant removal should be considered especially in young and active patients who report credible irritation and limited ROM following operative treatment. Overall our results provide advance of function and decrease of pain levels following implant removal. Therefore, in case of patients’ explicit request and credible limitation or pain during daily activities, we suggest implant removal to be performed after fracture consolidation. Future comparative randomized controlled trials are needed to confirm the substantial need for overall need of implant removal after plate osteosynthesis of dislocated clavicle fractures.

Limitation of this study is the low patient count (27 patients). Also the lack of a control group can be considered as limitation. Strength of this study is the prospective study design including functional outcome of 27 consecutive patients who explicitly requested implant removal after treatment of dislocated clavicle fractures.

## Conclusion

Pain as the main reason followed by irritation during daily activities and sports could be identified as risk factors for patients’ wish for implant removal. Based on our results with clearly improved functional outcome and overall decreased pain levels, we consider implant removal after plate osteosynthesis of the clavicle as a beneficial operative intervention.

## Abbreviations

ROM: Range of motion
MSQ: Munich shoulder questionnaire
SPADI: Shoulder pain and disability index
DASH score: Disabilities of the arm, shoulder and hand score
Constant score: Constant-Murley shoulder outcome score
IR: Implant removal
Modified constant score: Modified constant-Murley shoulder outcome score
n.s.: Not statistical significant
ORIF: Open reduction-internal fixation
s.: Statistical significant
